# Electrochemical Promotion of CO Oxidation on Na-Promoted Pt/YSZ: Interaction Between Multiple Promoting Species

**DOI:** 10.1007/s11244-018-0896-3

**Published:** 2018-01-12

**Authors:** Efstratios Stavrakakis, Danai Poulidi

**Affiliations:** School of Chemistry and Chemical Engineering, Queen’s University Belfast, Stranmillis Road, Belfast, BT9 5AG UK

**Keywords:** Electrochemical promotion, CO oxidation, Na promotion, Multiple promoters

## Abstract

The combined promotional effect of electrochemically-supplied O^2−^ and chemically-supplied Na^+^ promoters, was studied for the case of CO oxidation on Pt/YSZ. Four different sodium coverages (0.16, 1.6, 8 and 40%) were loaded onto the catalyst surface and the catalytic behaviour was compared with a nominally ‘clean’ catalyst under a wide range of reactants’ ratios under open-circuit and polarised conditions. Sodium generally increased oxygen adsorption by lowering the work function of the catalyst. However, sodium promoted the catalytic rate only at coverages up to 1.6% and worked synergistically with O^2−^ promoting species to an increased overall promotion of the catalytic rate. At higher sodium coverages, i.e. θ_Na_ ≥ 8%, the catalytic behaviour was strongly affected by the interactions between the sodium species, the catalyst, the reactants and oxygen ions promoting species. The postulated formation of stable sodium oxide species on the catalyst pores reduced the active catalytic area which resulted in poisoning the catalytic rate and suppressing EPOC effect, respectively. It is suggested that these stable sodium oxide species which also induced a permanent EPOC effect by oxygen storage, were formed by the migrated oxygen ions.

## Introduction

Electrochemical promotion of catalysis (EPOC) is a phenomenon where the application of small currents or potentials causes significant activity and selectivity modification of catalysts supported on ionic or mixed ionic-electronic conductors [1]. Several studies have shown that EPOC is due to electrochemically controlled migration (spillover or backspillover) of promoting species from the conducting support to the catalyst surface and vice versa with the direction depending on the applied current or potential [2]. The promoter migration on the gas-catalyst interface results in the formation of an overall neutral double layer which changes the catalyst work function, and modifies by this way the catalyst ability to bind with each of the reactant molecules. The induced changes in the catalyst work function by low promoter coverage have be correlated as:

1 $$\Delta \Phi ={\text{e}}\Delta {{\text{U}}_{{\text{WR}}}}$$

where ΔΦ is the variation of catalyst work function and ΔU_WR_ is the change of the potential between the catalyst and the reference electrode [3]. Depending on the catalytic rate, r, response to changes of catalyst potential, ΔU_WR_ (or ΔΦ), there are four types of behaviours at constant reactive conditions [4]:

- Electrophobic, when ∂r/∂Φ > 0.
- Electrophilic, when ∂r/∂Φ < 0.
- Volcano, when r passes through a maximum with varying ΔU_WR_.
- Inverted volcano, when r passes through a minimum with varying ΔU_WR_.

In EPOC, promoters are supplied to the catalyst electrochemically, in situ and reversibly. The latter is the main difference between electrochemical and conventional chemical promotion of catalysts where only ex situ addition of promoters can take place, during the catalyst preparation. Despite of this operational difference, the promotional function in both cases is identical and the phenomena are relied on the same promotional rules [5]. Generally, electropositive promoters (e.g. Na^+^) increase the chemisorption of reactants that behave as electron acceptors and decrease this of electron donors while for electronegative promoters (e.g. O^2−^) this case is reverse [1]. Electropositive promoters such as alkali metals have been extensively used in chemical promotion of catalysts [6]. Addition of low alkali coverage results in a sharp decrease in the work function of transition metal catalysts (e.g. Pt) while for higher coverages of up to 100%, the work function smoothly increases [1, 6, 7]. This change of behaviour was explained by the ionic form that alkalis exist in low coverage which changes the electronic properties of the catalyst atoms in vicinity. By increasing alkali coverage, the alkali overlayer obtains a more metallic character and the alkali-surface bond length increases [1, 6, 8]. In the case of CO oxidation which is the probe reaction in this study, oxygen is the electron acceptor and CO which is amphoteric adsorbent, acts as the electron donor. Previous studies that investigated the Li-promotion of CO oxidation showed that the catalytic rate was enhanced in rich-CO conditions for a 20% Li-coverage on Pt while the reaction was poisoned for any reactants ratio at higher Li-coverage on the catalyst [9]. Similar observations were reported for CO oxidation on electrochemically Na-promoted Pt and the critical Na-coverage was 2%, since larger alkalis have higher ionisation potential and induce the minimum work-function at lower coverages [10].

Although EPOC and chemical promotion are very similar research areas, they were investigated in parallel for many years and very few studies have engaged both of them. The initial attempts included the use of EPOC in order to define the best promoters and the optimum coverage for chemical promotion applications [11, 12]. The first actual study that combined EPOC and chemical promotion was reported by Pliangos et al. [13] which found that promoting species supplied electrochemically and chemically can act synergetic and increase even more the performance of a catalyst. Ibrahim et al. [14, 15] studied more thorough this case and tried to correlate the coverage of pre-existing sodium on the catalyst and this of the oxygen promoting species induced by EPOC, with the overall effect on the catalytic rate of ethylene oxidation on Pt/YSZ. They concluded that for low sodium coverage (i.e. θ_Na_ ≤ 1%) on the catalyst, the two promoting species synergistically promoted the reaction, while at high sodium coverage there was a significant interaction between the oxygen and sodium promoting species.

The present study will investigate further the case of electrochemical promotion of a catalyst pre-covered by several levels of sodium, using as a probe reaction the CO oxidation on Pt/YSZ. The aim is to study the combined promotional effect by Na^+^ and O^2−^ promoters and the complexity of the interactions between these promoters on electropromoted catalytic systems. The fact that this reaction is well studied both on the specific electrocatalytic system via EPOC [16–19] and on alkali-promoted Pt through chemical [9, 20, 21] or electrochemical promotion [10, 22], will assist for more secure conclusions.

## Materials and Methods

### Catalyst System Preparation

The electrocatalytic system used in this work was a three-electrode Pt/YSZ/Au pellet, consisting of a platinum catalyst film also acting as the working electrode, an yttria-stabilised zirconia (YSZ) pellet (an oxygen ion conducting solid electrolyte) and gold counter and reference electrodes. The solid electrolyte was fabricated by 8% mole YSZ powder supplied by Praxair. A pellet (20 mm in diameter, 2 mm in thickness) was formed by uniaxially pressing 2.2 g of YSZ powder under 4 tons for 30 s by a hydraulic press (Specac). The pellet was sintered then at 1500 °C for 12 h and the final dimensions after densification were 15 mm in diameter and 1.5 mm in thickness. Both sides of the cylindrical pellet were polished by 400 grit silicon carbide sandpaper to assist electrodes adhesion.

The Pt catalyst film was fabricated by brush-painting one side of the pellet with ca. 10 mg of Pt paste (5542, ESL) and sintering at 850 °C for 30 min. On the other side of the pellet, Au paste (A118, Metalor) was brush-painted to prepare counter and reference electrodes and finally sintered at 800 °C for 30 min.

Sodium (in the form of a NaOH solution) was gradually added onto the catalyst surface at the end of each set of kinetic and electrocatalytic experiments. A micropipette was used for the deposition of 5 μl NaOH aqueous solutions of increasing concentrations and the catalyst pellet was then dried for 1 h at 350 °C in nitrogen flow. The % coverage of sodium species on the Pt surface was calculated as follows. SEM analysis indicated an average Pt particle size of around 1.5 μm, so for 10 mg total mass of the catalytic film and considering spherical geometry for the particles, the platinum surface area was estimated 19 cm^2^ using the following equation:

2 $$SA=\frac{{6~ \cdot m}}{{D \cdot \rho }}$$

where SA is the platinum surface area, m is the platinum mass, D is the particle size, ρ is the platinum density [23]. Previous studies [24, 25] indicated that full coverage of the Pt film needs 10^15^ Na atoms per cm^2^. The % coverage was then calculated by the amount of sodium added during each deposition step. Table 1 shows the NaOH solutions used in each case and the calculations for sodium % coverage on the catalyst surface. It should be mentioned that the values in this table are cumulative since sodium deposition is irreversible after each step. Five different levels of sodium coverage, θ_Na_, on Pt were used: ‘nominally clean’ (without any sodium addition), 0.16, 1.6, 8 and 40%.

**Table 1:** Values of sodium loading and calculation of sodium coverage percentage on platinum catalyst

NaOH (M)	volume (μl)	Na (atoms)	Na/Pt (atoms/cm^−2^)	Coverage %
1 × 10^−5^	5	3 × 10^13^	0.02 × 10^14^	0.16
1 × 10^−4^	5	3 × 10^14^	0.16 × 10^14^	1.6
5 × 10^−4^	5	1.5 × 10^15^	0.79 × 10^14^	8
2.5 × 10^−3^	5	7.5 × 10^15^	3.96 × 10^14^	40

XRD, SEM and EDX analysis were conducted on the ‘nominally clan’ pellet prior to any experiment and on the catalyst pellet with 40% of sodium coverage on the catalytic surface (used 40% Na–Pt/YSZ) at the end of all kinetic and electrocatalytic experiments. Post-op XRD analysis (data not shown here) confirmed that no changes in the crystalline structure of the YSZ pellet and the Pt catalyst occurred during the experiments conducted in this work. The SEM/EDX results will be discussed in Sect. 3.

### Experimental Set-Up

A single chamber “EPOC type” reactor [1] was used in this research where all the electrodes were exposed in the same atmosphere. This type of reactor has been reported [26] to function as a continuous-stirred tank reactor (CSTR) for the applied total flowrate (200 ml/min) and atmospheric pressure. The reactor consists of a cylindrical quartz tube with 50 cm^3^ volume and has an open and a closed end. The open end was sealed by a series of stainless steel Swagelok fittings to allow for gas inlet and outlet and electrical connections. The Pt/YSZA/Au pellet was suspended inside the reactor by the gold wires attached to each electrode with the use of a ceramic (macor) clip. The reactor was placed vertically inside a tubular furnace (VCTF1, Vecstar) equipped with an 8-segment Eurotherm controller; in addition, an independent K-type thermocouple was used to measure the temperature near the catalyst pellet.

The reacting gases were 20% CO in N_2_, 99.9995% N_2_ and air supplied by BOC. Regarding to the analysis unit, the outputs of this system were: the CO_2_ concentration in the reactor outlet stream, the temperature inside the reactor, the applied overpotential (or open circuit potential) and the induced current flow. For measuring the actual reaction temperature, the K-type thermocouple was connected to a digital thermometer (KM340, Comark). The production of CO_2_ was measured by a Vaisala GMP343 CO_2_ analyser connected to the reactor outlet. In order to apply constant potential between the working and the reference electrode and measure the current flow between the working and the counter electrode, a potentiostat (EmStat) was connected by crocodile clips attachment on the tips of gold wires at the top of the reactor. The readings of the CO_2_ concentration (ppm) and current, i, or potential, V_WR_ were recorded for a five-second interval by a computer with the use of the instrument’s software.

### Electrocatalytic Experiments

The conditions inside the reactor were the same for all the experiments, i.e. the pressure inside the reactor was atmospheric, the temperature was kept at 350 °C and the total flowrate of the gas mixture was 200 ml/min (STP). The same cycle of experiments was repeated for all catalyst % sodium coverages reported here. Each experiment involved different ratio between the reactants and followed the same protocol in order to maintain continuity and obtain directly comparable results between the range of conditions employed in this work. According to this protocol, the potentiostatic (chronoamperometric) catalytic transients included the following steps: (i) open-circuit for 90 min, (ii) application of + 1 V for 60 min, (iii) application − 1 V for 60 min, (iv) open-circuit for 90 min, (v) application − 1 V for 60 min and (vi) open-circuit for 90 min. Regarding to the sequence within a cycle of experiments, first the partial pressure of CO, p_CO,_ was kept constant at 0.5 kPa and this of O_2_ varied from 0.35 to 10 kPa. Then, the partial pressure of oxygen was kept constant at 0.5 kPa and the range of CO partial pressures, p_CO,_ was varied from 0.25 to 5 kPa. Nitrogen flow was used overnight between consecutive tests. The open-circuit catalytic rate values presented in the results section correspond to the steady-state values of the initial open-circuit step. In addition, during the chronoamperometric sequence followed, there were two steps with negative polarisation. Only the second negative polarisation step was taken into account for the corresponding effect on the catalytic rate, while the use of the first negative overpotential application (step iii), was to assist discharging and restriction of permanent EPOC observed during preliminary experiments.

In order to quantify the observed promotional effect, the reaction rate enhancement ratio, ρ, and faradaic efficiency, Λ, were calculated as the two parameters that describe the magnitude of electrochemical promotion [1]:

3 $$\rho =\frac{r}{{{r_o}}}~~~~~$$

4 $$\Lambda =\frac{{\Delta {\text{r}}}}{{I/2{\text{F}}}}~~~$$

where r is the catalytic rate under EPOC effect, r_o_ is the catalytic rate under open-circuit conditions, Δr is the difference in the catalytic rate because of polarisation, I is the current density and F is the Faradays constant. Depending on the polarisation (positive or negative), the individual parameters ρ_(+1V)_ and ρ_(−1V)_ were used herein to express the corresponding rate enhancement. The rate enhancement ratio corresponding to the effect of Na was designated as ρ_Na_ and defined as:

5 $${\rho _{{\text{Na}}}}~=\frac{{{r_{{\text{Na,o}}}}}}{{{r_{\text{o}}}}}$$

where r_Na,o_ is the catalytic rate promoted by Na and r_o_ is the catalytic rate of a ‘clean’ catalyst (both rates are under open circuit conditions). Finally, ρ_total_ is the parameter that express the overall catalytic rate enhancement ratio caused by combined EPOC and Na-promotion compared to a Na-free catalyst under O.C. and similar reactive conditions:

6 $$\rho_{\text{total}}=\frac{{{r_{{\text{Na}}, \pm }}}}{{{r_o}}}$$

where r_Na,±_ is the catalytic rate upon sodium modification and polarisation.

## Results and Discussion

### (Na–)Pt/YSZ Characterisation

**Fig. 1:**
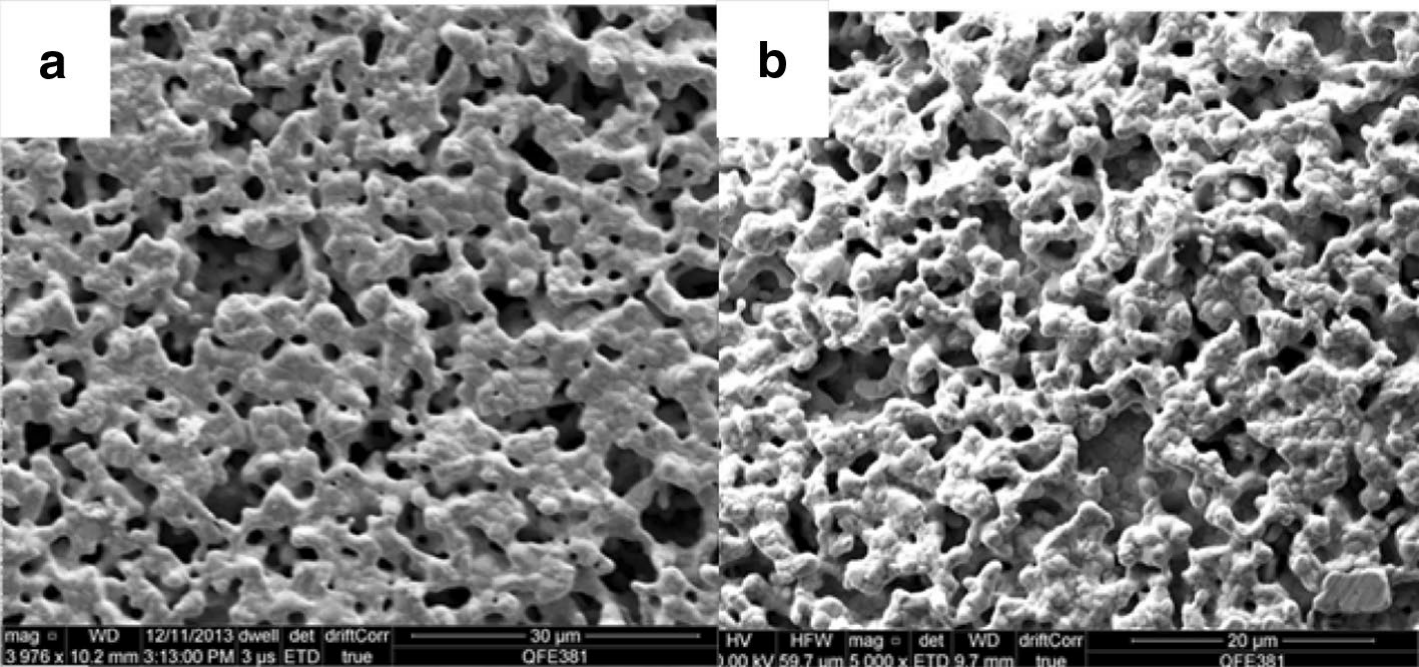
SEM images of **a** the fresh nominally “clean” Pt surface and **b** the used 40% Na-modified Pt surface

The morphology of the fresh nominally “clean” Pt surface is shown in the SEM images in Fig. 1. Figure 1a shows that the platinum film was continuous and porous. From SEM images at higher magnification (not shown here) the average Pt particle size was estimated to be around 1.5 μm. Overall, the morphology of the catalytic film met EPOC requirements for porosity and continuity. Adequate porosity would offer large three phase boundaries (tpb) area, where the promoting species responsible for EPOC are generated. Continuity of the film implies electrical continuity and is important for the polarisation experiments and the promoting species migration on the entire catalytic surface [1]. Figure 1b shows that there are only minor morphology changes on the Pt surface after 40% Na-modification and prolonged (over than four months) electrocatalytic experiments. Platinum particles size, pore size and distribution was similar to this of the fresh unmodified surface, suggesting that there was no sintering effect during these experiments. Also, sodium addition does not seem appear to have affected the morphology of the catalytic surface.

**Fig. 2:**
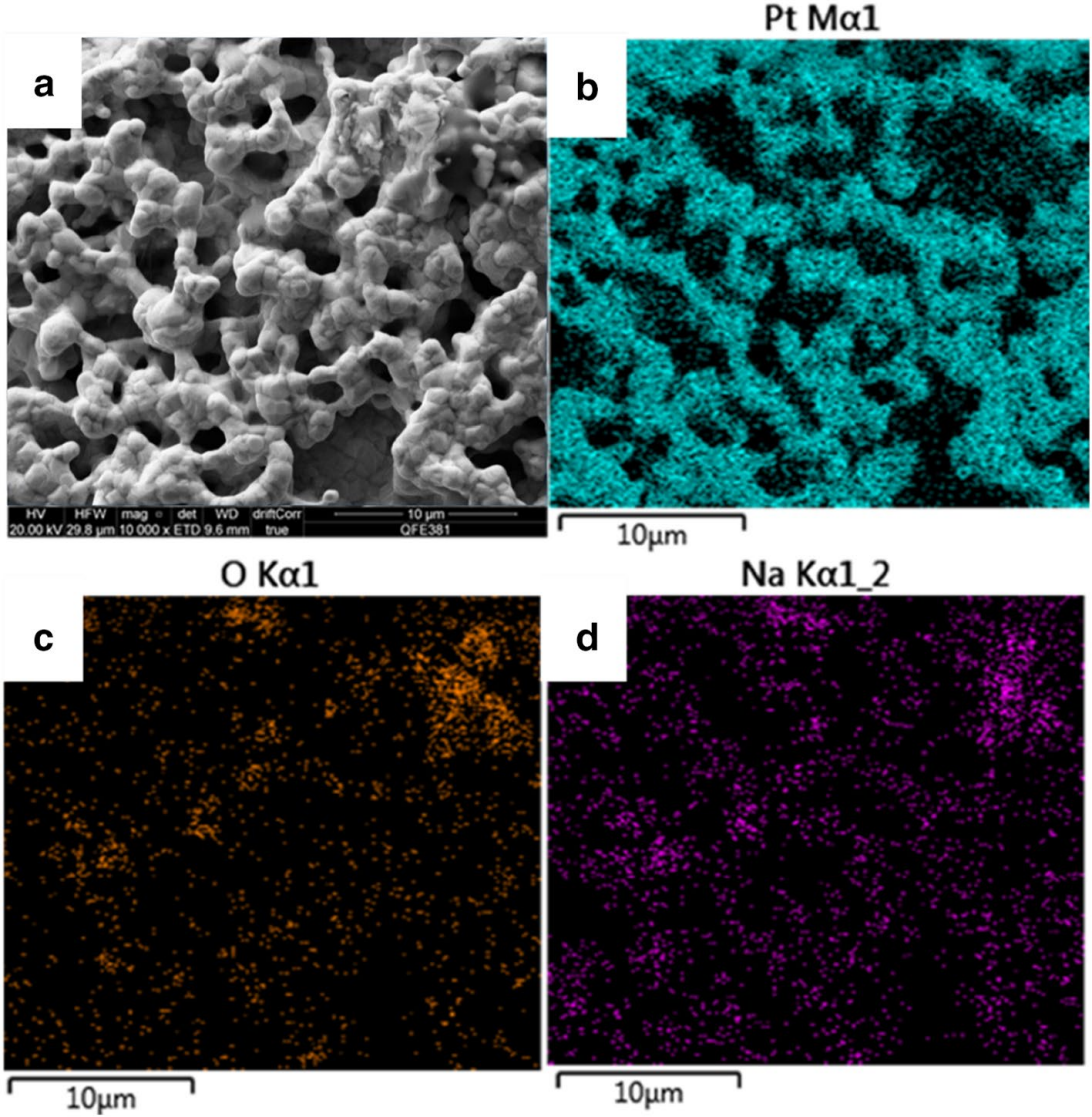
SEM image of the used Na-modified Pt surface with magnification of ×10,000 (**a**) and the corresponding X-ray mapping of Pt (**b**), O (**c**) and Na (**d**)

The elemental distribution mapping of the fresh nominally “clean” Pt surface was obtained through EDX (not shown here). No impurities were detected within the EDX detection limit and the quantification of the EDX spectra indicates that there was pure Pt in the surface. Elemental mapping was also obtained for the used 40% Na-modified Pt surface as shown in Fig. 2 where the SEM micrograph (a), the corresponding distribution of Pt (b), oxygen (c) and sodium (d) can be seen. As we can see, sodium shows increased density around and inside the catalyst film pores, suggesting preferential segregation of sodium species in these areas. Since sodium was supplied to the catalyst by the form of a liquid solution, it is very likely that high amount incorporated into the high porosity of the catalyst and accumulated in the catalyst-solid electrolyte interface. It is also interesting to note that oxygen showed identical atomic distribution to sodium i.e. high densities of oxygen were detected around or inside the catalyst film pores. This observation indicates that for this level of sodium coverage, sodium species may exist in the form of oxides or even superoxides form since a high oxygen/sodium mass ratio was recorded. This comes in agreement with the findings of Ibrahim et al. [27], who studied through XPS the same case, i.e. the addition of sodium on Pt films supported on a YSZ pellet. They reported that in a Na-modified Pt catalyst, sodium can exist in the form of sodium carbonate, oxide or hydroxide, especially for high sodium coverages, i.e. higher than 11%. In our work, the formation of carbonates was not confirmed, due to experimental limitations but their presence is very likely due to the experimental conditions employed (i.e. use of CO oxidation as the probe reaction).

### Open-Circuit Kinetics—Sodium Modification

**Fig. 3:**
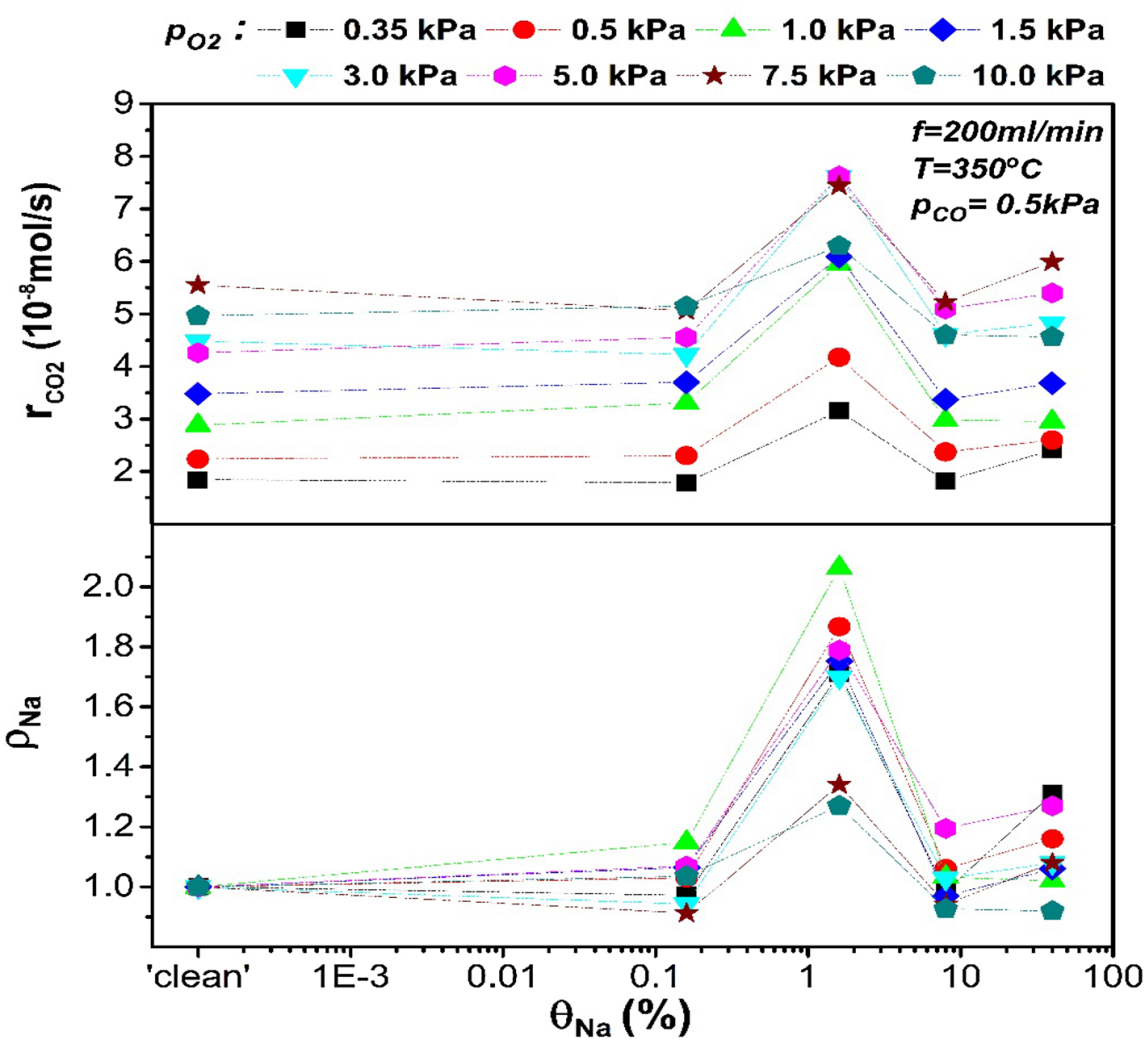
Open-circuit rates and the corresponding rate enhancement ratio ρ_Νa_ as a function of ${{\text{p}}_{{{\text{O}}_2}}}$ (at a constant p_CO_ = 0.5 kPa) for a range of sodium coverages. The experimental conditions are T = 350 °C, flowrate = 200 ml/min. The trend-lines are used as a visual aid only and do not correspond to an actual fit of the experimental data

**Fig. 4:**
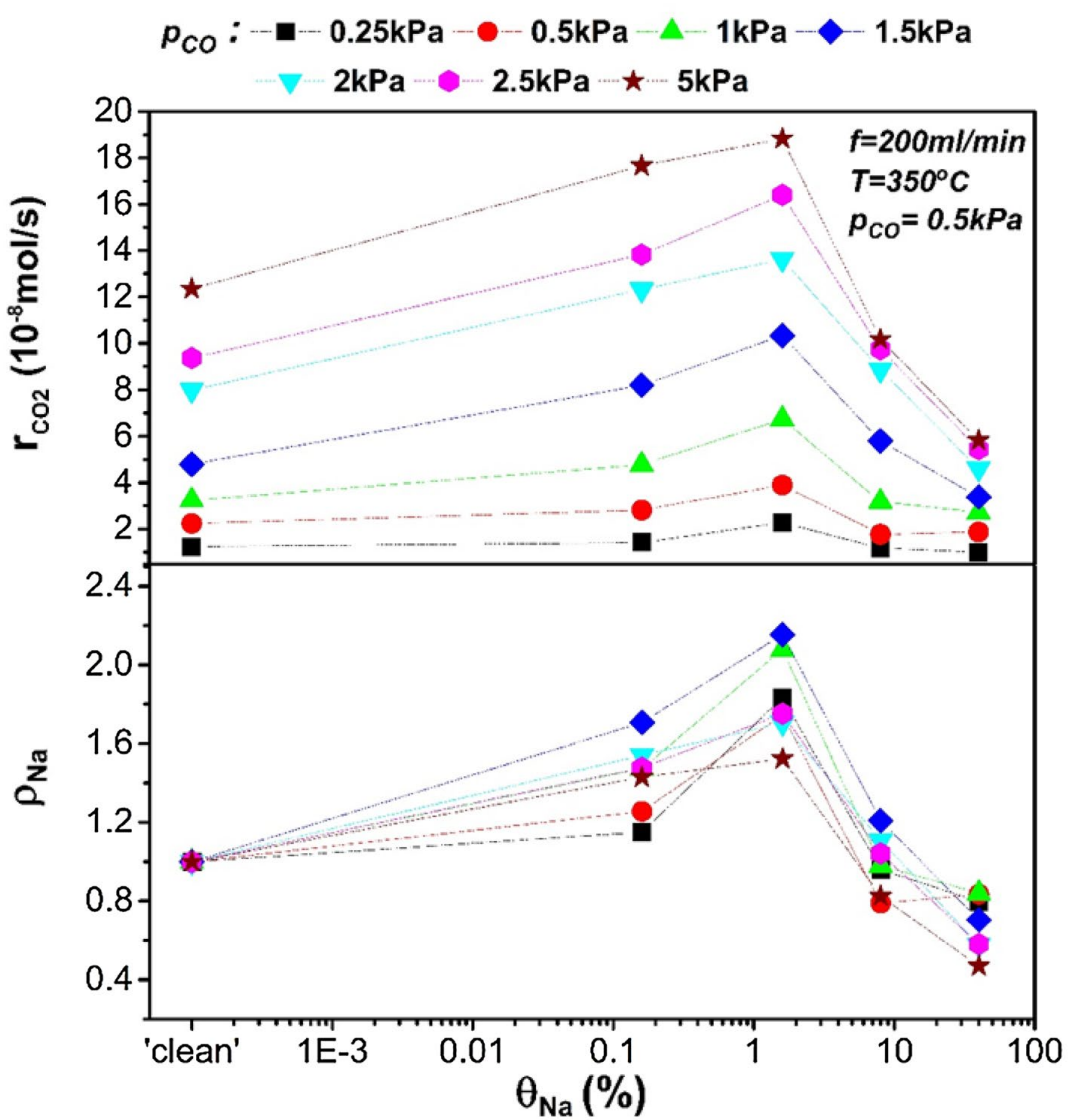
Open-circuit rates and the corresponding rate enhancement ratio ρ_Νa_ as a function of p_CO_ (at a constant ${{\text{p}}_{{{\text{O}}_2}}}$ = 0.5 kPa) for a range of sodium coverages. The experimental conditions are T = 350 °C, flowrate = 200 ml/min. The trend-lines are used as a visual aid only and do not correspond to an actual fit of the experimental data

Next, the effect of sodium as a promoter on the open-circuit catalytic rate of CO oxidation on Pt will be discussed. Sodium as a promoter, is known to decrease the work function of a catalyst [1, 6, 7]. In the context of catalytic adsorption that means that the chemisorption bond of the electron acceptor adsorbate will be strengthened while this of the electron donor adsorbate will be weakened [1, 6]. In the present case, sodium addition and thus work function decrease, is expected to increase the adsorption of oxygen (electron acceptor) and decrease the adsorption of carbon monoxide (electron donor). The induced changes in the catalytic rate, excluding any effect of active catalytic sites blocking due to Na addition, will depend on the reaction order. For example, if the reaction is positive order with respect to oxygen, sodium addition on the catalytic surface will be expected to enhance the catalytic rate. Figures 3 and 4 show the catalytic rate and rate enhancement ratios of the nominally “clean” and Na-modified catalyst under open-circuit conditions with respect to partial pressures of oxygen, ${{\text{p}}_{{{\text{O}}_2}}}$, and CO, p_CO_, respectively. The catalytic rate on a nominally “clean” Pt surface was positive order for low ${{\text{p}}_{{{\text{O}}_2}}}$ and zero order for higher ${{\text{p}}_{{{\text{O}}_2}}}$. For the case of increasing p_CO_, the reaction was positive order with p_CO_ for the range of CO partial pressures used in this work. Sodium coverage, θ_Na_, of 0.16% on Pt surface, enhanced the reaction rate mainly under rich CO conditions. On the other hand, the enhancement of the catalytic rate was limited for high oxygen partial pressures when the reaction was zero order to oxygen prior to sodium addition on the catalyst. This fact indicates that the promotional effect by this level of sodium modification was due to increased oxygen chemisorption on a predominantly CO-covered Pt surface where the kinetics were positive order in oxygen. Increasing sodium coverage to 1.6%, caused a general further promotion which was more pronounced at reactants ratio close to stoichiometry and moderately reducing conditions. A sodium rate enhancement ratio of ρ_Na_ ≈ 2.2 was obtained. This promotional effect is less pronounced under high oxygen partial pressures which suggests that 1.6% of sodium loading possibly enhanced further oxygen chemisorption. However, this statement will be further discussed later alongside to the kinetics upon polarisation.

With further sodium addition (i.e. 8% coverage), in oxidative conditions the catalytic rate returned to values similar to those measured for the nominally ‘clean’ catalyst while at CO-rich atmospheres a poisoning effect was observed. Poisoning was more extended for θ_Na_ = 40%, and reducing conditions but in excess of oxygen there was a slight promotion compared to the Na-free catalyst. Hence, we can conclude that the promotional effect peaked at 1.6% of sodium coverage while the propensity for this behaviour was more intense under near-stoichiometric conditions. Similar behaviour was reported for low CO excess by Yentekakis et al. [10] in CO oxidation on electrochemically Na-promoted Pt. Electrochemically supplied sodium ions increased the oxygen adsorption and enhanced the catalytic rate by a factor of 5 at a Na-coverage of 2% on Pt. Above this critical coverage, a poisoning effect was observed and attributed to formation of Na–CO–Pt and Na–O–Pt complexes which blocked the Pt surface at reducing and oxidising conditions, respectively. The decomposition temperature of these complexes during reactive conditions was reported to be ca. 400 °C [10]. Bertolini et al. [8] showed the formation of a similar CO–K–Pt complex during co-adsorption of CO and K on Pt, which decomposed at ca. 407 °C. Considering that (i) the employed temperature in these experiments was 350 °C (below the complex decomposition temperature), (ii) the poisoning effect was more pronounced at strong CO presence and (iii) that at high sodium coverages the interactions of sodium with the metal surface are stronger, the inhibition of the reaction could be associated to formation of similar complexes on the catalyst surface.

As discussed earlier, the SEM–EDX analysis of the catalytic surface with 40% of sodium coverage, showed that sodium was not distributed uniformly on the surface and there was a formation of Na–O islands around or inside the catalyst pores. According to the literature, sodium in high coverages obtains a metallic character and the interaction with the reaction components, i.e. the catalyst and the reactants, become stronger [6, 28, 29]. Although the form of the sodium species is not clear by the SEM–EDX surface characterisation presented earlier, the reaction kinetics suggest that the in-situ formation of Na–O–Pt and Na–CO–Pt complexes is very likely.

Nevertheless, the poisoning effect at high sodium coverage can be explained by a non-uniform distribution on the catalyst and possible accumulation of sodium species and formation of islands, as shown in the SEM–EDX analysis. It appears that the number of the catalytic sites activated by sodium was low with a concomitant decrease in the available active surface area because the sodium species were blocking the pores of the platinum film. Hence, the promotional effect by the sodium-induced work function changes, did not surmount the poisoning effect by the active catalytic sites blocking. On the contrary, for low sodium coverages, sodium has a strong ionic character which allows weaker interaction with the reactants or the surface and the distribution on the surface could be uniform [6]. Hence, the active sites blocking was less significant and thus the promotional effect was dominant under these conditions.

Comparing our results to the kinetics of the catalytic CO oxidation on Li-doped Pt [9], there are similarities in alkali-promotion behaviour. Yakovkin et al. reported that lithium coverages up to 20% slightly enhanced (ca. 10%) the reaction for ratios of CO/O_2_ partial pressures higher than 0.15, while higher Li-coverages induced a poisoning effect at any reactants ratio. A possible reason for the lower promotional effect and the higher critical alkali coverage reported in their work, in comparison the present study, is that larger alkalis can induce higher work function reduction at low coverages. Hence, sodium can induce higher promotion than lithium at lower coverages, thus avoiding the active sites blocking that is caused at higher alkali coverages [6, 9, 29].

### Effect of Sodium on EPOC

The combined promotional effect of sodium and oxygen ion promoting species on CO oxidation catalytic rate and the interaction between these promoters, will be discussed next. In general, the faradaic efficiency, Λ, values related to all potentiostatic catalytic rate changes discussed in this section were higher than unity, which is the condition of EPOC phenomenon existence. The catalytic rate changes upon polarisation was up to 40 times higher than the faradaic rate of oxygen ions supply to the catalyst. These values together with the rate enhancement ratios up to 1.63 (to be discussed here), constitute the EPOC effect as moderate [1].

**Fig. 5:**
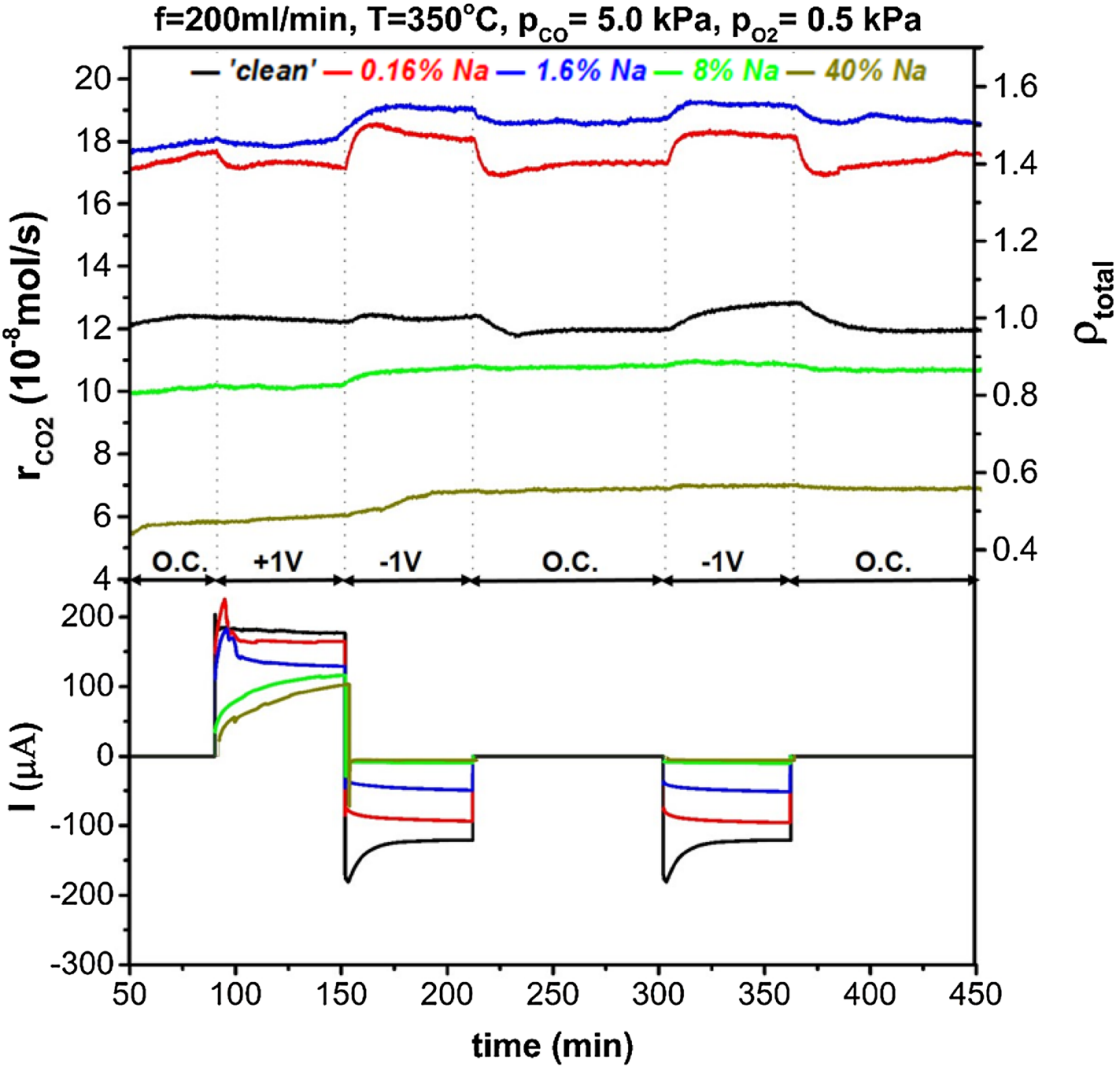
Dynamic reaction rate transients and corresponding current during potentiostatic measurements under reducing conditions (p_CO_ = 5 kPa, ${{\text{p}}_{{{\text{O}}_2}}}$ = 0.5 kPa) for a range of sodium coverages. On the top-right axis the total enhancement ratio, ρ_total_, compared to a ‘clean’ catalyst under open-circuit is shown

**Fig. 6:**
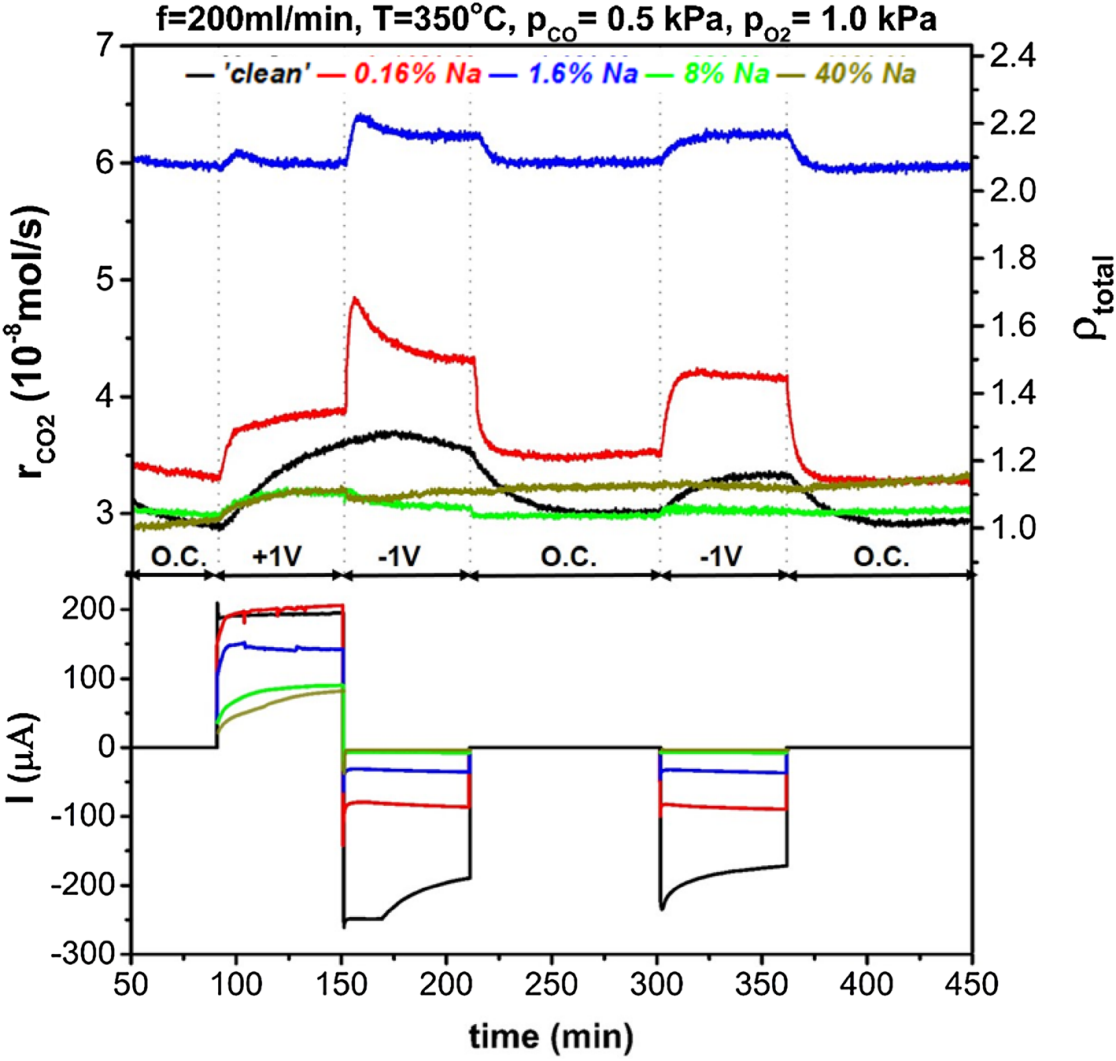
Dynamic reaction rate transients and corresponding current during potentiostatic measurements under stoichiometric conditions (p_CO_ = 0.5 kPa, ${{\text{p}}_{{{\text{O}}_2}}}$ = 1.0 kPa). On the top-right axis the total enhancement ratio, ρ_total_, compared to a ‘clean’ catalyst under open-circuit is shown

**Fig. 7:**
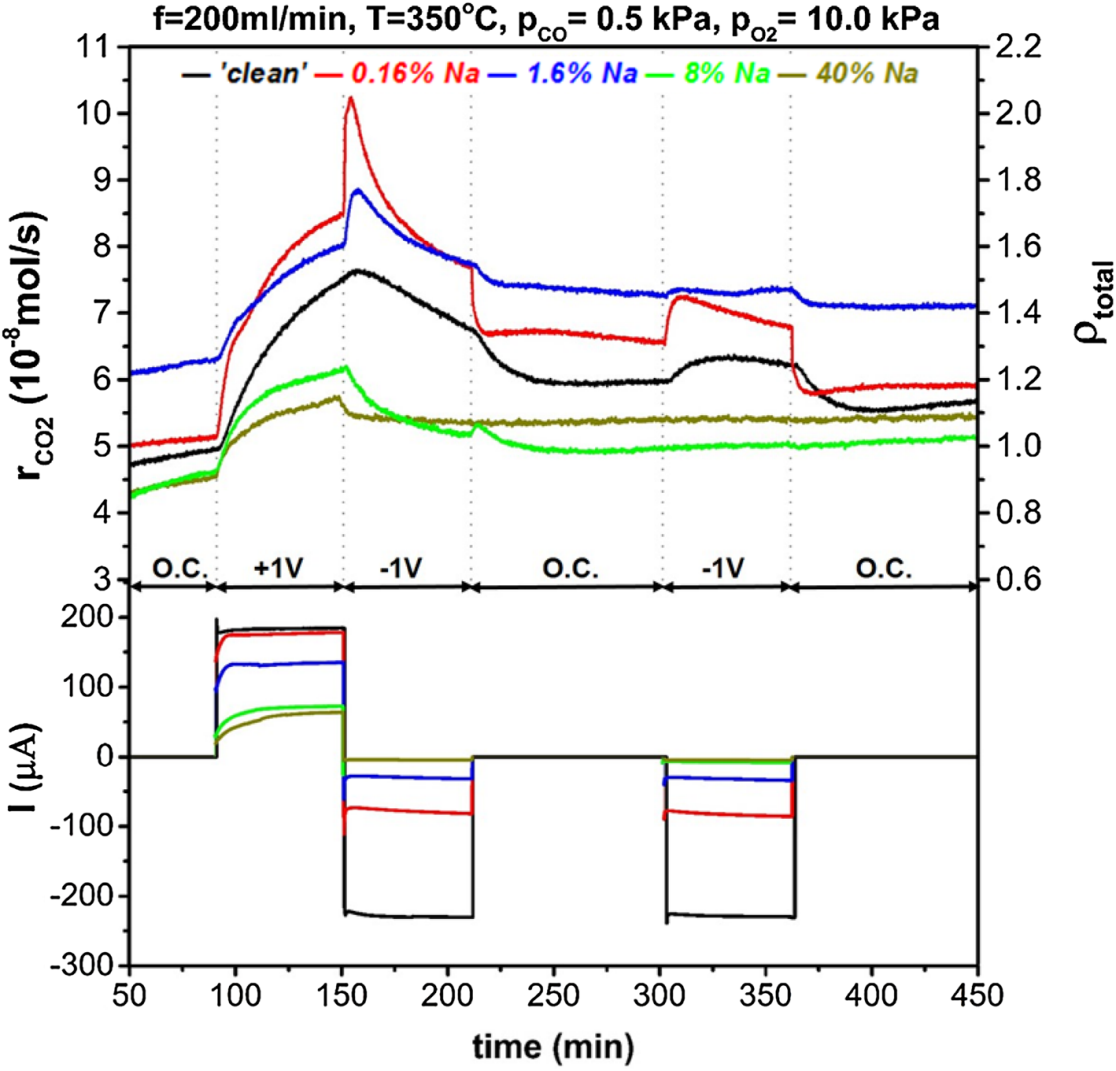
Dynamic reaction rate transients and corresponding current during potentiostatic measurements under hilghy oxidising conditions (p_CO_ = 0.5 kPa, ${{\text{p}}_{{{\text{O}}_2}}}$ = 10 kPa) for a range of sodium coverages. On the top-right axis the total enhancement ratio, ρ_total_, compared to a ‘clean’ catalyst under open-circuit is shown

Figures 5, 6 and 7 show the reaction rate potentiostatic (chronoamperometric) transients under different sodium coverage and reacting conditions. Application of positive overpotential causes migration of oxygen ions from the solid electrolyte to the three-phase boundaries and oxygen promoting species generated there, would spillover the catalyst and favour CO (electron donor) chemisorption by strengthening the bond between CO and the catalyst. On the contrary, the reverse effect is expected for negative overpotential which would strengthen the bond between the catalyst and oxygen (i.e. electron acceptor) and enhance oxygen chemisorption [4].

In Fig. 5 we can see the reaction rate transients for highly reducing atmospheres, i.e. p_CO_ = 5 kPa, ${{\text{p}}_{{{\text{O}}_2}}}$ = 0.5 kPa. For the nominally ‘clean’ catalyst, the reaction followed an electrophilic behaviour which became more pronounced at low sodium coverage, i.e. θ_Na_ ≤ 1.6%. Higher sodium modification, gradually poisoned the open-circuit catalytic rate and restricted the EPOC effect, as well. By increasing the partial pressures ratio of O_2_ to CO in the reacting gas feed, the reaction obtained an inverted volcano character for a nominally “clean” catalyst, as shown in Fig. 6. Sodium coverage of 0.16% enhanced EPOC effect under negative polarisation, while for 1.6% coverage the observed promotion under positive overpotential was restricted. Higher sodium coverages suppressed any rate promotion at negative overpotentials and turned the reaction’s behaviour to electrophobic. Figure 7 shows chronoamperometric transients for highly oxidising conditions. The catalytic rate is promoted under both positive and negative polarisation (inverted volcano behaviour) for the nominally “clean” catalyst. In this case, there was a clear indication of permanent/persistent EPOC effect on the intermediate open-circuit step despite the use of an anterior negative polarisation stage. This phenomenon has been attributed to storage of O^δ−^ promoting species at the catalyst-YSZ interface and is more enhanced under oxidizing conditions [30–32]. During the final open-circuit state, the permanent enhancement was eliminated since the catalytic system had been subjected to further negative polarisation. Sodium coverage of 0.16% generally enhanced EPOC, while θ_Na_ of 1.6% enhanced “electrophobicity” of the reaction by minimizing promotion under negative potential. For higher θ_Na_, promotion took place only for positive potential and the permanent EPOC effect was more pronounced.

The permanent EPOC effect not only became stronger for oxidising conditions, but it was generally observed at any reactants ratio for high sodium coverages. Specifically, as shown in Fig. 6, under stoichiometric conditions, the reaction was also permanently promoted after positive overpotential interruption, but to a lower extent. Interestingly, under reducing conditions (Fig. 5) a small permanent EPOC effect occurred after negative overpotential interruption. The explanation of this behaviour may be two-fold: (i) the permanent promotional effect has been attributed to oxygen ions storage at the catalyst–electrolyte interface and (ii) sodium at high coverages obtains a metallic character and bounds strongly with oxygen. Regarding to the latter, a previous study of Ibrahim et al. suggested that oxygen storage is enhanced by high sodium coverages on Pt/YSZ, after application of positive potential [15, 27]. In addition, the SEM–EDX analysis showed that sodium at high coverages, accumulated, possibly in the form of sodium oxides, inside the catalyst film pores. These observations suggest that these sodium oxides were generated with spillover or backspillover oxygen ions and were responsible for the observed permanent EPOC effect. Similar alkali oxides/superoxides induced a permanent EPOC effect in a study by de Lucas-Consuegra et al. [33], where a K-β′Al_2_O_3_ solid electrolyte was used. It was shown by in-situ (cyclic voltammetry, FTIR) and ex-situ techniques (SEM–EDX) that potassium oxides were formed and stabilised on Pt, after electrochemically pumped potassium ions reacted with adsorbed oxygen. The presence of sodium carbonates and oxides on the catalyst surface has also been confirmed by XPS analysis conducted by Ibrahim et al. [27] in a previous study.

**Fig. 8:**
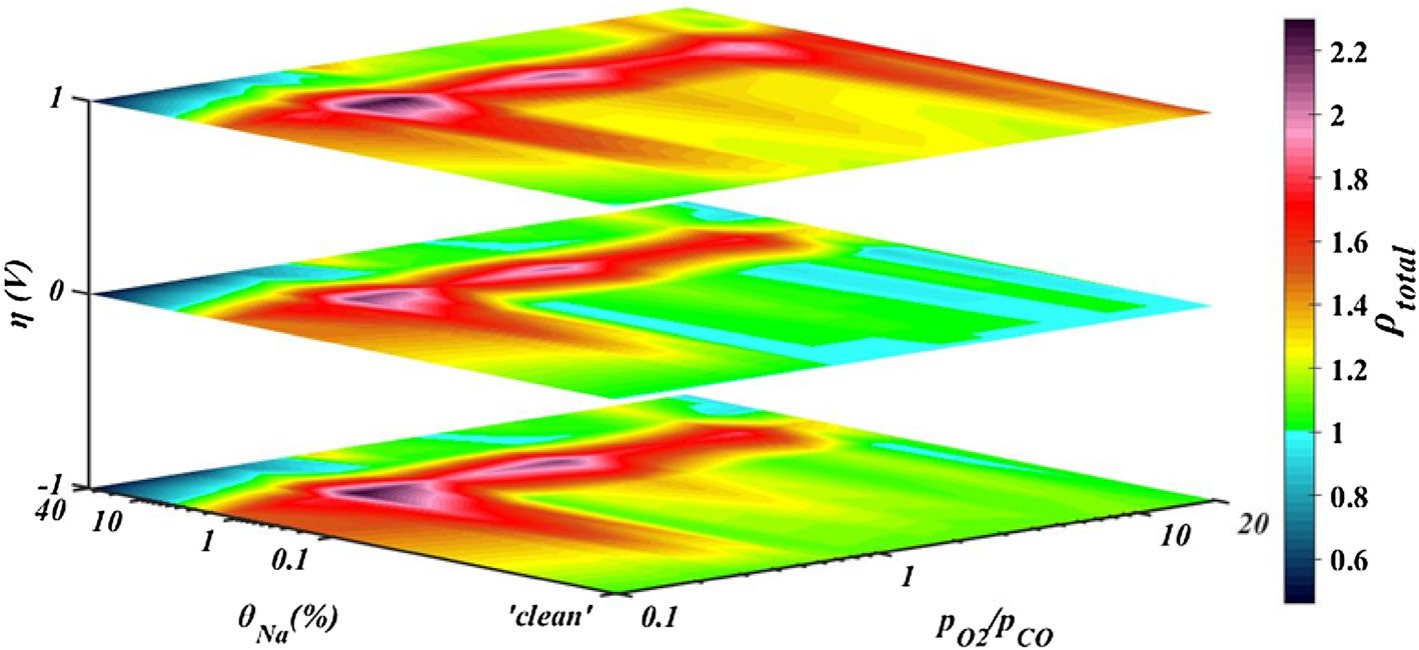
Contour map of the overall effect of combined sodium chemical promotion and oxygen ion electrochemical promotion on the catalytic rate of CO oxidation on Pt. The values on the colormap correspond to the total enhancement of the catalytic rate upon polarisation and sodium modification compared to the open-circuit rate for a ‘clean’ catalyst

The overall effect of combined sodium chemical promotion and oxygen ion electrochemical promotion on the catalytic rate of CO oxidation as observed during the potentiostatic transients for all reactants ratios tested, is summarised in Fig. 8. The total enhancement, ρ_total_, of the catalytic rate with respect to the open-circuit rate of a nominally “clean” catalyst (Eq. 6) is shown. As such, ρ_total_ in open-circuit, η = 0, corresponds to Na-promotion, i.e. is the ratio between catalytic rate of Na-modified catalyst and this of the nominally “clean” catalyst at a fixed ${{{{\text{p}}_{{{\text{O}}_2}}}} \mathord{\left/ {\vphantom {{{{\text{p}}_{{{\text{O}}_2}}}} {{{\text{p}}_{{\text{CO}}}}}}} \right. \kern-0pt} {{{\text{p}}_{{\text{CO}}}}}}$ ratio (Eq. 5). Additionally, the total enhancement for the ‘‘clean’’ catalyst equates to the reaction rate enhancement due to polarisation (Eq. 3).

As can be seen in Fig. 8, the rate enhancement under positive polarisation, ρ_(+1V),_ for the nominally “clean” catalyst increased with the oxygen partial pressure (i.e. increased oxygen coverage); thus, positive overpotential that increases the adsorption strength of CO, gradually enhanced the promotional effect. For highly reducing conditions application of positive potential which favours CO adsorption, had a slightly poisoning effect on the reaction since the catalyst was saturated by CO. The maximum ρ_(+1V)_ observed was 1.5 and corresponded to the highest ${{{{\text{p}}_{{{\text{O}}_2}}}} \mathord{\left/ {\vphantom {{{{\text{p}}_{{{\text{O}}_2}}}} {{{\text{p}}_{{\text{CO}}}}}}} \right. \kern-0pt} {{{\text{p}}_{{\text{CO}}}}}}$ ratio. Negative polarisation generally promoted the reaction on the nominally “clean” catalyst but to a lower extent. Under near-stoichiometric conditions, the reaction showed inverted volcano behaviour, which indicates that both the reactants were weakly adsorbed on platinum. The lowest enhancement, ρ_(−1V),_ recorded for the highest ${{\text{p}}_{{{\text{O}}_2}}}$ when the catalyst had already adsorbed the highest amount of oxygen.

Strong overall promotion (up to 230%) was achieved at low sodium levels where sodium and oxygen promoting species acted synergistically. At 0.16% sodium coverage, higher overall promotion was observed by imposition of positive overpotential in oxidising conditions. On the contrary, enhancement upon negative overpotential increased for moderate CO-conditions, compared to the enhancement for a ‘clean catalyst’. It was observed that sodium coverage of 0.16% strengthened the character of the reaction that existed in the “clean” catalyst. Ibrahim et al. [15] reported a similar effect by 0.11% sodium coverage on Pt for electrochemical promotion of ethylene oxidation. They observed that low sodium coverage enhanced EPOC effect without altering significantly the open-circuit rate suggesting that there is an interaction between the sodium promoting species and the electrochemically supplied O^2−^ species. Surface characterisation techniques (i.e. SEM, XPS) conducted by Ibrahim et al. [15, 27] showed that sodium species existed in a form of carbonates, hydroxides or oxides at coverage lower than 1% on platinum which blocked part of the catalyst active sites. These sodium species were described to be unstable and decomposed upon polarisation, generating new sodium species that induced further enhancement after EPOC activation. Thus, the effect of θ_Na_ < 1% on ethylene oxidation which was the probe reaction in Ibrahim’s study and was electrophilic at any reactants ratio tested, was to magnify this behaviour [15]. In the present study, CO oxidation presented a varying character upon potential changes, depending on the reactants ratios, i.e. electrophilic at highly reducing conditions and inverted volcano at any other ratio. The intriguing feature is that sodium promoting species apparently enhanced the pre-existed behaviour of the reaction, which suggests that similarly to Ibrahim’s study [15] new catalytically active sites were possibly created after EPOC activation and decomposition of unstable sodium species.

For 1.6% of sodium on the catalyst surface, the combined promotion was strong for reducing conditions regardless of polarisation while for oxidising conditions the largest enhancement was observed upon positive overpotential. The electrochemical promotional effect at 1.6% θ_Na_, was restricted possibly due to decreased values of current (as shown in Figs. 5, 6, 7) for this sodium coverage. Sodium species might have blocked some of the tpb sites where the oxygen charge transfer reaction occurs, as a previous study indicated [14]. Upon negative polarisation, the rate was not modified significantly for increased CO partial pressures while the enhancement after application of + 1 V overpotential was becoming more pronounced for increasing oxygen concentration. Pliangos et al. [13] reported a similar effect on the rate of CO oxidation on 1% Na–Pt/YSZ under high oxygen partial pressure. While for a “clean” catalyst the reaction was inverted volcano, sodium caused high chemical promotion and the behaviour under EPOC effect was altered to electrophobic [13].

At high sodium coverage (≥ 8%), the reaction rate changes were determined by the strong interactions of sodium species with the catalyst, the reactants and also with oxygen ion promoters. A strong oxygen adsorption was counterbalanced by the speculated formation of stable sodium species which poisoned the catalytic rate and suppressed EPOC effect. Hence, a weak promotion effect was observed only for positive overpotential imposition while the reaction was generally poison in reducing conditions.

**Fig. 9:**
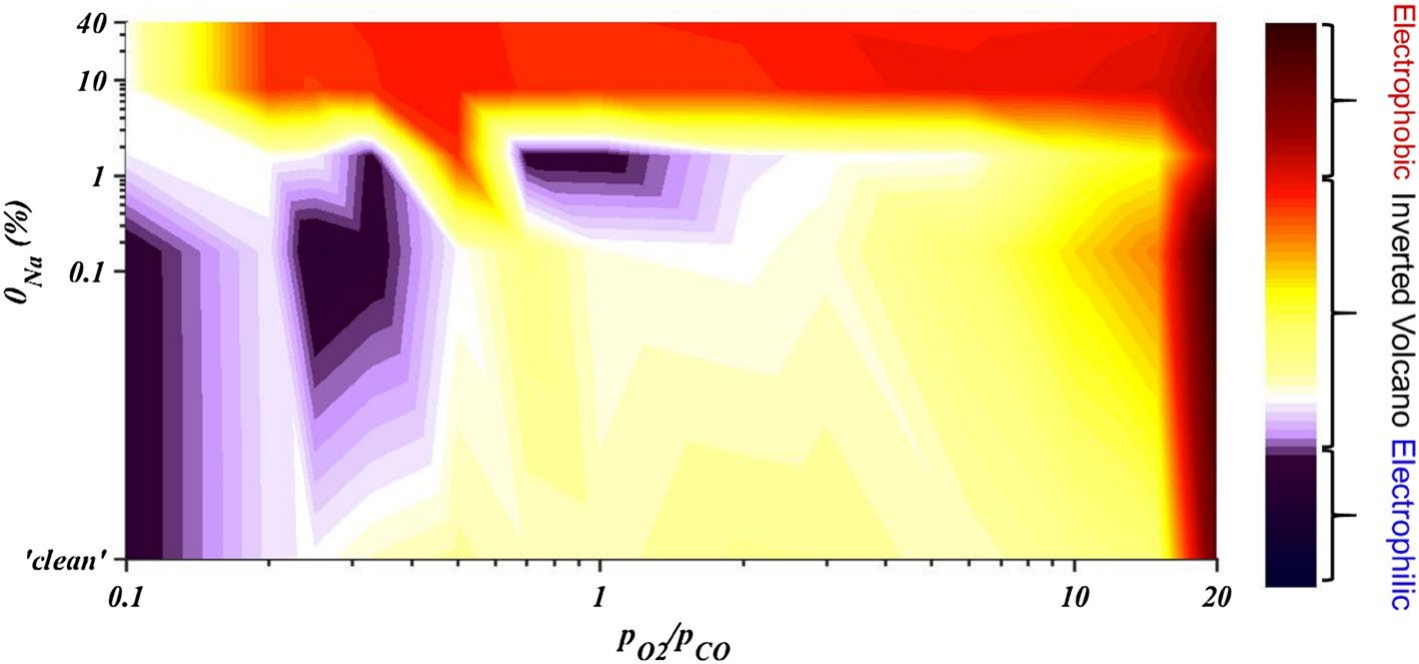
Contour map of reaction’s behaviour upon polarisation for various sodium modifications and reactants ratio. This map does not include any actual rate enhancement ratios and is simply used as a schematic representation of the type of behaviours observed in potentiostatic transients

Figure 9 shows a contour map summarising the behaviour of the reaction after EPOC activation with respect to sodium coverage and reactants’ ratio. It should be mentioned that this map does not include any actual values but it is just a schematic representation of the type of behaviours identified during the potentiostatic experiments. Three different types of behaviour were observed: electrophilic, inverted volcano and electrophobic. At low partial pressures of oxygen and low sodium coverage, oxygen is weakly adsorbed while CO is strongly adsorbed and the reaction had an electrophilic behaviour. Under the same conditions, the reaction turned into inverted volcano by increasing sodium coverage as oxygen adsorption becomes stronger while this of CO weakens. At high oxygen partial pressures, the reaction change behaviour from inverted volcano to electrophobic at lower sodium modification, since the bond between the catalytic surface and oxygen was relatively stronger. In all other cases, the reaction presented an inverted volcano behaviour, since the adsorption of both CO and oxygen was weak. High sodium coverages expanded the electrophobic character of the reaction towards the full range of reacting conditions by strengthening oxygen adsorption.

## Conclusions

CO oxidation on Pt/YSZ with combined O^2−^ and Na^+^ promoting species was studied. Sodium promoting species increased the oxygen chemisorption by lowering the work function of the catalyst. Low sodium coverages, i.e. up to 1.6% promoted significantly the catalytic rate when the kinetics were positive order in oxygen. At the same time, the relatively unstably sodium species worked synergistically with the electrochemically supplied oxygen ions promoting species. Especially for the coverage of 0.16%, the reaction’s behaviour upon EPOC was enhanced by the possible formation/decomposition of sodium species during polarisation and creation of new active catalytic sites. At high sodium coverages (θ_Na_ ≥ 8%) on the catalyst, a large increase in the oxygen adsorption was induced and changed the reaction behaviour upon polarisation to electrophobic at any reactants ratio. However, the interaction of sodium with the catalyst, the reactants and oxygen ions promoting species became significant as well. Stable sodium oxide species (and possibly other stable complexes) were formed inside and around the catalyst pores and blocked part of the active catalytic area and the catalyst-solid electrolyte interface. This resulted in the apparent poisoning of the catalyst thus suppressing EPOC effect. Sodium oxide species were possibly formed by the migrated oxygen ions during polarisation and induced a permanent EPOC effect at any reactants ratio, by oxygen storage. The case of the multiple promoters and their accompanied interactions, is worth to be investigated in more complex reactions to examine the effect on the selectivity of such reactions.
